# Poculum Caritatis—The Cup of Kindness

**Published:** 1906-12-01

**Authors:** 


### POCULUM CARITATIS—THE CUP OF KINDNESS.

One seldom reads of Christmas catastrophes due to over-eating or over-drinking among the rich. On the other hand, it is common to find in the Press during Christmas-time accounts of persons having "eaten themselves to death." The "Poculum Caritatis"—the Cup of Kindness—is denied to the poor of modern times on the ignorant pretext that it is bad for them. There is weird cruelty in administering to those poor ones, who cannot now share the joys of former Christmases, huge quantities of unaccustomed food without at the same time adding what is especially essential—something to aid its digestion. Such cruelty is the more poignant at the time of Christmas, when the mind is full of memories of a brighter past. We have heard it gravely suggested that the stimulating condiments of mustard and pepper act as well in promoting the digestion of roast beef and plum-pudding as brandy, and that the pauper population had not, even in these last days, been denied a free use of the mustard-pot with their Christmas dinner. The mustard-pot is surely a poor substitute for the "Poculum Caritatis"—the Cup of Kindness—whose presence at the Christmas feasts of the past added a sense of cheer to the lives of the poor who were so hospitably invited to share them.

